# Challenges of breast cancer screening: Number 9 – September 2023

**DOI:** 10.1055/s-0043-1775931

**Published:** 2023-10-16

**Authors:** Alexandre Vicente de Andrade, Clécio Ênio Murta de Lucena, Danielle Chambô dos Santos, Eduardo Carvalho Pessoa, Fabio Postiglione Mansani, Felipe Eduardo Martins de Andrade, Giuliano Tavares Tosello, Henrique Alberto Portella Pasqualette, Henrique Lima Couto, Jose Luis Esteves Francisco, Rodrigo Pepe Costa, Sandra Regina Campos Teixeira, Thaís Paiva Moraes, Agnaldo Lopes da Silva Filho

**Affiliations:** 1 Pontifícia Universidade Católica de São Paulo, São Paulo, SP, Brazil; 2 Universidade Federal de Minas Gerais, Belo Horizonte, MG, Brazil; 3 Faculdade de Medicina da Santa Casa de Misericórdia de Vitoria, Vitória, ES, Brazil; 4 Universidade Estadual Paulista, Botucatu, SP, Brazil.; 5 Universidade Estadual de Ponta Grossa, Ponta Grossa - PR, Brazil; 6 Hospital Sírio-Libanês, São Paulo, SP, Brazil; 7 Universidade do Oeste Paulista, Presidente Paulista, SP, Brazil.; 8 Centro de Pesquisas da Mulher Rio de Janeiro, Rio de Janeiro, RJ, Brazil.; 9 Clínica Redimama-Redimasto de Belo Horizonte, Belo Horizonte, MG, Brazil.; 10 Faculdade de Medicina de São José do Rio Preto, São José do Rio Preto, SP, Brazil; 11 Hospital de Base do Distrito Federal, Brasília, DF, Brazil; 12 Pontifícia Universidade Católica de São Paulo, Campinas, SP, Brazil; 13 Rede Mater Dei de Saúde, Belo Horizonte, MG, Brazil; 14 Universidade Federal de Minas Gerais, Belo Horizonte, MG, Brazil

## Key points

- Mammography is the method of choice for breast cancer screening and the only one that demonstrates a reduction in mortality in the population at usual risk.
- The frequency of performing and the age at which mammogram screening begins are a controversial topic in the literature. Data in our country point to a significant portion of breast cancer in women under 50 years of age.
- The Brazilian Federation of Gynecology and Obstetrics Associations (Febrasgo), the Brazilian Society of Mastology (SBM) and the Brazilian College of Radiology and Diagnostic Imaging (CBR) agree that mammogram screening should be performed annually by all women from 40 years old.
- In Brazil, there is an unequal distribution of mammography devices in different regions. Screening policies must consider this inequality.
- The vast majority of services in Brazil perform opportunistic screening for breast cancer. The implementation of screening organized by age group and risk stratification can optimize the costs of the public health system.
- High-risk patients need to be screened differently from usual-risk patients. These patients need to have access to breast magnetic resonance imaging (MRI) and start screening at an earlier age.
- The abbreviated MRI protocol for breast cancer screening of high-risk patients may improve their adherence and access to the screening program.
- Breast ultrasound is not a screening method in isolation. However, it plays a role as a complementary method to mammography and MRI in specific scenarios, and replaces MRI in patients with contraindications to the use of this method.
- Dense breasts have low sensitivity for screening mammography.

## Recommendations

- Mammography should be performed as the preferred method of breast cancer screening for women at usual risk.
- The subgroup of women between 40 and 50 years of age at usual risk should preferably be evaluated with annual mammography, given the prevalence of breast cancer in this age group in Brazil.
- Ultrasonound should not be used as an isolated method in the screening scenario, but always complementary to mammography or MRI of the breasts. Ultrasound can also be used in patients with contraindications to MRI (allergy to contrast, incompatibility with the device, claustrophobia, presence of a pacemaker or other implanted device).
- Screening of high-risk patients should be done with annual MRI and mammography. When it is not possible to access the MRI exam, ultrasound can be used with reservations related to the examiner’s experience in the breast ultrasound exam.
- The abbreviated protocol for breast cancer screening of high-risk patients should be considered in services that perform breast MRI, as it saves time and has shown to be equally effective in published series. Furthermore, this protocol can increase patients’ adherence to an annual screening program.
- The implementation of organized screening should be encouraged in breast cancer screening services, since this measure optimizes program costs.
- The subgroup of patients with dense breasts should be evaluated with caution, considering the low sensitivity of mammography. In these patients, complementary ultrasound can be performed, as well as MRI screening for patients at normal risk and dense breasts (considering the availability of this resource in the various regions of the country).
- Patients at intermediate risk for breast cancer should be evaluated individually for the proposed screening. Some of these patients, especially those with dense breasts or lesions that increase the risk, may be candidates for annual MRI in addition to mammography.

## Background

Breast cancer is the most prevalent in Brazil and the leading cause of cancer death among women. As the chance of cure is higher than 95% if diagnosed early, breast cancer screening measures are fundamental. Technological advances in diagnostic methods, such as digital mammography, tomosynthesis and MRI associated with the evolution in drug treatment for breast cancer are identified as the cause of the drop in breast cancer mortality in developed countries. However, breast cancer mortality curves continue to rise in most regions in Brazil, as a reflection of the lack of access to diagnosis and treatment. These data should make us reflect on the challenges of screening, which go beyond the correct use of available methods and include health policies and public management, allowing the early detection of a lesion, its diagnosis and treatment, so that the screening results in reducing mortality from breast cancer. At least 70% of the target population must be involved for an effective cancer screening program, and these numbers reach a maximum of 35% of women in Brazil. Several factors contribute to this scenario, such as: difficult access to the exam (although the number of mammography devices in Brazil is sufficient, they are very unevenly distributed); fear of performing the exam (even with several awareness campaigns, especially the Pink October, information is often not clear, simple and direct as it should be); and, above all, the fact that screening is done opportunistically in Brazil, depending on whether the patient seeks the physician, without active tracking of patients. There are many challenges to the success of a screening program. The first step is to ensure the quality of the image and the mammogram report, which can be achieved through mammogram quality programs. The second step is to ensure quick access to the diagnosis of the suspicious mammographic finding through biopsy. And, finally, provide adequate treatment, avoiding delays and providing access to the most effective drugs. Investments in a more effective screening program to address these issues are high, but are also cost-effective. The diagnosis of initial lesions allows treatment to be de-escalated, whether surgical (more conservative surgeries) or adjuvant (less use of chemotherapy and radiotherapy), consequently increasing the chances of cure for patients. 1 2

## What is the starting age for mammogram for breast cancer screening and how often should mammography be performed for women at usual risk?

According to CBR, SBM and Febrasgo recommendations published in 2012 and updated in 2017, breast cancer imaging screening by age group should occur as follows:

- Women under 40 years old – usual risk; in general, mammography is not recommended in this age group;
- Women between 40 and 74 years old – all women in this age group should have a mammogram on an annual basis, preferably using the digital technique (category A); in places where breast tomosynthesis is available, it should preferably be used;
- Women over 75 years old – screening, preferably with the digital technique, is recommended on an individual basis in this age group; women with a life expectancy greater than seven years and who may undergo cancer treatment, considering their comorbidities, should continue mammographic screening (category D).

## What is the starting age for mammogram for breast cancer screening and how often should mammography be performed in high-risk women?

According to the CBR, SBM and Febrasgo recommendations published in 2012 and updated in 2017, guidelines for the subgroup of high-risk patients are:

- Women with a mutation in BRCA1 or BRCA2 genes or first-degree relatives with a proven mutation should undergo annual mammogram screening from the age of 30 (category B);
- Women with a lifetime risk ≥ 20% calculated by one of the mathematical models based on family history should start 10 years before the age of diagnosis of the youngest relative (not earlier than 30 years of age) (category B);
- Women with a history of having undergone chest irradiation between ages of 10 and 30 years should undergo annual mammogram screening from the eighth year after radiotherapy treatment (not earlier than 30 years of age) (category C);
- Women diagnosed with genetic syndromes that increase the risk of breast cancer (such as Li-Fraumeni, Cowden and others) or affected first-degree relatives should undergo annual mammogram screening after diagnosis (not earlier than 30 years of age) (category D).

Note that personalized mammogram screening is being discussed more and more nowadays. Before starting this screening, it is important that the patient has her risk assessment carried out by the professional assisting her. If it is a high-risk patient, the screening program should be intensified. Clearly, the future is to adjust screening for these populations. 1 2

### Categories

- Category A – Recommendation based on strong scientific evidence, with uniform consensus between CBR, SBM and Febrasgo in strong support of this recommendation.
- Category B – Recommendation based on reasonable scientific evidence, with uniform consensus between the CBR, SBM and Febrasgo in strong support of this recommendation.
- Category C – Recommendation based on little scientific evidence, but with consensus between the CBR, SBM and Febrasgo in strong support of this recommendation.
- Category D – Recommendation based on consensus of experts from CBR, SBM and Febrasgo in support of this recommendation.

## What is the role of ultrasound in breast cancer screening?

Breast echography or ultrasound has the challenges of its quality and the expertise of the examiner. This method is not used alone in breast cancer screening neither for usual-risk nor high-risk patients. It can be used in addition to mammography and MRI and to guide biopsies in case of suspicious lesions. 2 In the scenario of screening high-risk patients, ultrasound can be used in places without access to breast MRI, and/or if there is any contraindication to this exam (allergy to contrast, incompatibility with the device, claustrophobia, presence of a pacemaker or other implanted device). The major limitation of ultrasonography is its high false-positive rate and consequently, the need to perform biopsies. This is a highly operator-dependent method with increased effectiveness if performed by a professional experienced in the method and knowledgeable about breast imaging and its nuances in various imaging methods. 2 The cost-effectiveness of performing ultrasound should be analyzed before proposing the use of this method. High breast density can decrease the sensitivity of mammography by 30-48%, as breast cancer is normally radiodense. Furthermore, breast density itself is an independent risk factor for breast cancer. Even though advances were obtained with digital mammography and tomosynthesis, increasing sensitivity from 55% to 70% (digital x conventional), some cancers may still not be detectable in the midst of dense breast parenchyma. In these cases, a complementary exam is recommended for patients with dense breasts at usual risk, and ultrasound is considered the complementary modality of choice. Supplemental screening with ultrasound is an option to increase cancer detection in women with dense breasts at intermediate risk. 2

## What is the role of MRI in screening high-risk populations?

High-risk patients are usually a group of younger patients and consequently, with denser breasts. In this specific subgroup, the use of mammography alone has low sensitivity. Since breast MRI is a functional method and not purely morphological, it does not depend on breast density for its effectiveness. Therefore, the use of MRI in high-risk patients is more effective, alone or in combination with mammography. 3 4 5 Breast MRI in high-risk women has shown greater sensitivity than mammography as a screening method. The combination of mammography and MRI in this population has greater sensitivity (92%) than MRI alone. Furthermore, combined MRI and mammography are more sensitive (92.7%) than combined ultrasound and mammography (52%). Therefore, MRI is recommended annually in high-risk women. Screening high-risk women with breast MRI is cost-effective and the cost-effectiveness of MRI screening increases with increasing risk of breast cancer, i.e., the greater the risk of the studied population the greater the positive predictive value and specificity of the method. 5

## Final considerations

An optimized screening program is essential to reduce breast cancer mortality in Brazil, plus it is cost-effective. All women should have their breast cancer risk assessment at age 30 to ensure they do not belong to a minority classified as high risk. Every asymptomatic woman at usual risk should undergo annual mammography/tomosynthesis starting at 40 years of age, as studies indicate a reduction in mortality from breast cancer due to early diagnosis, which also offers better surgical treatment options and more effective systemic treatment. Regarding the age to stop screening, the patient’s clinical conditions, comorbidities and life expectancy should be considered. Particularly in patients aged 75 years or older who will undergo breast cancer screening. We should discuss the possibility of recall for repeat exams or even to perform additional exams, the risk of undergoing unnecessary (benign) biopsies, the risk of an overdiagnosis (diagnosis of a cancer that might never manifest itself clinically), and finally, the anxiety generated by screening. The risks and benefits of screening for each woman should not be discussed in general, but on an individual basis. For high-risk patients and in some special conditions, another complementary diagnostic method (MRI or ultrasound) should be considered.

National Specialized Commission on Breast Imaging of the Brazilian Federation of Gynecology and Obstetrics Associations

President:

Eduardo Carvalho Pessoa

Vice-President:

Henrique Lima Couto

Secretary:

Clecio Enio Murta de Lucena

Members:

Alexandre Vicente de Andrade

Danielle Chambô

Fabio Postiglione Mansani

Felipe Eduardo Martins de Andrade

Giuliano Tavares Tosello

Hélio Sebastião Amâncio de Camargo Júnior

Henrique Alberto Portella Pasqualette

Jorge Villanova Biazús

José Luis Esteves Francisco

Rodrigo Pepe Costa

Sandra Regina Campos Teixeira

Thais Paiva Moraes

## References

[1] Urban L A Schaefer M B Duarte D L Santos R P Maranhão N M Kefalas A L Recomendações do Colégio Brasileiro de Radiologia e Diagnóstico por Imagem, da Sociedade Brasileira de Mastologia e da Federação Brasileira das Associações de Ginecologia e Obstetrícia para rastreamento do câncer de mama por métodos de imagem Radiol Bras 2012 45 6 334 9 10.1590/S0100-39842012000600009

[2] Urban L A Chala L F Bauab S P Schaefer M B Santos R P Maranhão N M Recomendações do Colégio Brasileiro de Radiologia e Diagnóstico por Imagem, da Sociedade Brasileira de Mastologia e da Federação Brasileira das Associações de Ginecologia e Obstetrícia para o rastreamento do câncer de mama Radiol Bras 2017 50 4 244 9 10.1590/0100-3984.2017-0069 29036752 PMC10316951

[3] Kuhl C K Baltzer P You get what you pay for: breast MRI screening of women with dense breasts is cost-effective J Natl Cancer Inst 2021 113 11 1439 41 10.1093/jnci/djab120 34585246 PMC8562968

[4] Kuhl C K Abbreviated breast MRI for screening women with dense breast: the EA1141 trial Br J Radiol 2018 91 1090 2.0170441E7 10.1259/bjr.20170441 PMC6350487 28749202

[5] Kuhl C K Abbreviated Magnetic Resonance Imaging (MRI) for breast cancer screening: rationale, concept, and transfer to clinical practice Annu Rev Med 2019 70: 501 19 10.1146/annurev-med-121417-100403 30691370

